# Physical Activity as a Strategy to Reduce the Risk of Osteoporosis and Fragility Fractures

**DOI:** 10.5812/ijem.3309

**Published:** 2012-06-30

**Authors:** Magnus Karl Karlsson, Bjorn Erik Rosengren

**Affiliations:** 1 Clinical and Molecular Osteoporosis Research Unit, Department of Clinical Sciences, Lund University, Department of Orthopaedics, Skane University Hospital, Malmo, Sweden

**Keywords:** Osteoporosis, Physical Activity, Athletes

## Abstract

Childhood and adolescence are critical periods for the skeleton. Mechanical load has then been shown to be one of the best stimuli to enhance not only bone mass, but also structural skeletal adaptations, as both contributing to bone strength. Exercise prescription also includes a window of opportunity to improve bone strength in the late pre- and early peri-pubertal period. There is some evidence supporting the notion that skeletal gains obtained by mechanical load during growth are maintained at advanced age despite a reduction of physical activity in adulthood. The fact that former male athletes have a lower fracture risk than expected in their later years does not oppose the view that physical activity during growth and adolescence is important and it should be supported as one feasible strategy to reduce the future incidence of fragility fractures.

## 1. Introduction

The skeleton is a metabolically active organ that responds to mechanical stimuli by initiating or inhibiting bone modelling and remodelling in order to keep peak strains within a safe physiological range. Up to 40 % of the variance in bone strength is also estimated to be determined by the mechanical load that it supports (1). The feedback system, where the skeletal response depends on the characteristics of the load, is usually referred to after the mechanostat, a theory popularized by Harold Frost (2). Key features for osteogenic stimuli include a load that is dynamic, has a high magnitude, a high frequency and unusually distributed strains (3-5). The required mechanical load necessary to stimulate osteogenesis also decreases as the strain magnitude and frequency increases (3-5). But the osteogenic response to mechanical load becomes saturated after a few loading cycles (6). Following this, any additional load provides limited further benefit (7). However, bone cell mechanosensitivity recovers following rest so that separating loading into short bouts with periods of rest in between optimises the osteogenic response to loading (8-10). For example, four hours of rest between loads doubles the osteogenic response and the sensitivity to loading is almost completely restored after eight hours of recovery, as shown in animal studies (11). That is, the loading characteristics most beneficial for bone strength are very specific, making a general prescription of physical activity for cardiovascular health or weight reduction non-optimal for skeletal health.

There are currently many studies that have shown exercise to be associated with higher bone mineral density (BMD) (12-29) and lower fracture incidence (30-32) than expected by age and gender. The osteogenic response are also maturity and gender dependent (18, 33) so that the strongest response to mechanical stimuli occurs during growth, especially in the pre- or early pubertal period (12-29, 33-35). The response in adults is lower, in that physical activity can only reduce age related bone loss or at best produce increments in BMD of just a few percentage points (36, 37). These benefits are of questionable clinical significance for fracture reduction. The lower fracture incidence in physically active elderly people is therefore, probably the result of non-skeletal effects such as; increased muscle strength and/or improved neuromuscular function, traits which are possible to influence by training even in the tenth decade of life (38). Training in the elderly should therefore be structured to reduce the risk of falling, more than to prevent osteoporosis, if the aim is to reduce the incidence of fragility fractures (39).

## 2. Objectives

The objective of this review is to evaluate the skeletal effects of exercise through physical activity during growth and if exercise induced skeletal effects are retained with associated fracture reduction at older ages. This report is not a structured review, it is a review that primarily includes prospective controlled trials, preferably randomized studies found in PUBMED, that evaluate the skeletal effects of interventions with physical activity in the earlier years of life and the long term effects of exercise at reduced activity levels.

## 3. Exercise and the Skeleton in Athletes

The relationship between physical activity and an increase in BMD during the first two decades in life was reported 40 years ago when Nilsson et al. found that athletes had higher BMD than controls (40). This view has been supported in a variety of further articles that found that high impact sports such as tennis, squash, gymnastics, ice-hockey, volleyball and soccer are associated with a higher than expected BMD, while the practices of endurance sports such as; running, cycling and swimming showed less promising results (41). For example, in young female gymnasts, BMD has been shown to increase 30 to 85 % more rapidly than in sedentary children (42), but young tennis players display a 10-15 % arms side-to-side difference in BMD (18, 43). The difference is also more pronounced if the exercise is initiated before, rather than after puberty (18, 43). That is, much of our knowledge as regard to the adaption of the skeleton when exposed to increased mechanical load has been achieved from studies on athletes. However, these results provide us with information on what is possible, rather than what is probable, to reach by physical activity in children.

## 4. General Physical Activity and the Skeleton at Growth

**Figure 1:**
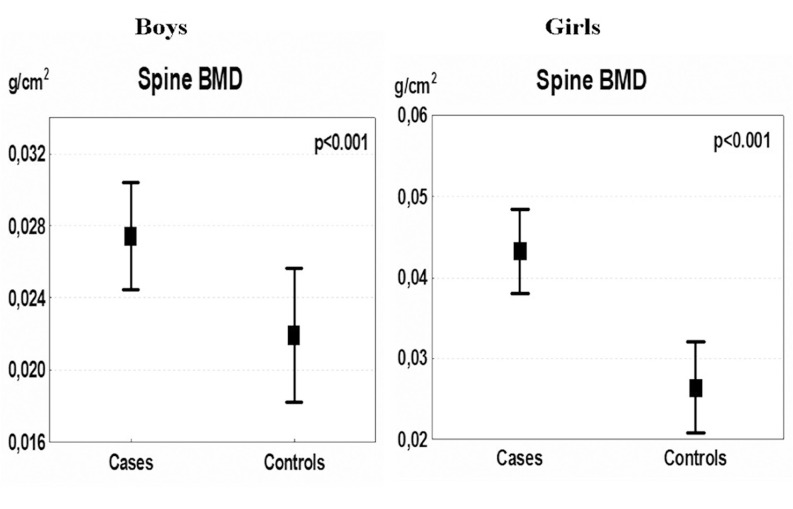
Mean Annual Changes in Lumbar Spine Bone Mineral Density (BMD) in Boys and Girls during the 5-year Intervention Presented as Means with 95 % Confidence Intervals

Studies in children with a moderate general level of physical activity have shown that exercise intervention is associated with skeletal benefits, but this is of lower magnitude than in athletes (12-29, 33-35) (Table 1). These benefits should, however, not be underestimated as even a small increase in bone mass can generate a more than two-fold increase in bone strength (8, 9, 44). Most intervention studies in pre- and peri-pubertal children utilize extra physical education classes or supplementary exercise in addition to physical education classes and most studies are short-term of less than 12 months duration (13, 15-17, 19, 20, 24, 25, 28, 29, 34, 45). But, there are now a few reports of up to a 5 year follow-up period that infer that long term moderate to intense interventions on a population based level may provide beneficial skeletal effects (14, 20-23, 26, 46) (Figure 1). These interventions can also be initiated without inducing an increased rate of childhood fractures (14, 21), an adverse effect of physical activity that has been postulated to occur when increasing the rate of trauma during periods of intense levels of physical activity (47-49). The reason for the maturity and sex-dependent response to mechanical load (18, 33) is postulated to be the result of exercise preferentially affecting surfaces of the bone undergoing apposition and hormonal discrepancies (50). The pre-pubertal skeleton also seems to have the capacity to respond to loading by adding more bone on the periosteal surface than would normally occur through growth-induced periosteal apposition (43, 51, 52). Although studies also infer that an endosteal apposition exists in pre-pubertal boys as a response to mechanical load (13, 51, 53), whereas such a response seems less likely in pre-pubertal girls (43, 53). Exercise in late puberty is associated with bone apposition on the endosteal surface, as shown in female tennis players (43), and the enlargement of bone size in response to loading has been reported to increase from pre- to peri-puberty in male, but not in female tennis players (43, 51). These differences confer in general a more beneficial response in boys than in girls.

**Table 1:** Skeletal Response Changes in Bone Mineral Content in Randomized and Non-Randomised Prospective Controlled Exercise Intervention Studies in Children and Adolescents.

Reference	Age of Participants	Type of Exercise Intervention	Duration of Intervention	Increase Higher in Cases *v.s* Controls
**Prepubertal (Tanner stage I)**				
Fuchs, *et al* (2001) (15)	99 children	High-impact jumping 10 minutes 3 times a week	7 months	BMC a: +4.5 % FN a, +3.1 % LS
				BMD a: +2.0 % LS a
	7.6 ± 0.2 years			BA a: +2.5 % FN a
Petit, *et al* (2002) (27)	68 girls	High impact 10-12 minutes 3 times a week	7 months	No effect
	10.0 ± 0.6 years			
MacKelvie, *et al* (2001) (22)	70 girls	High impact 10-12 minutes 3 times a week	7 months	No effect
	10.1 ± 0.5 years			
McKay, *et al* (2000) (24)	144 children	Moderate impact 10-30 minutes 3 times a week	8 months	BMD a: +1.1 % Tr a
	6.9 - 10.2 years			
Bradney, *et al* (1998) (13)	38 boys	Weight-bearing 30 minutes 3 times a week	8 months	BMD a: +1.2 % TB a, +2.8 % LS, +5.6 % legs,
				vBMD a: +5.6 % FM a
	10.4 ± 0.2 years			CT a: +6.4 % legs
Valdimarsson, *et al*. (2006) (28)	103 girls	Daily school physical educational classes	12 months	BMC a: +4.7 % LS a, 16.0 % Tr
				BMD a: 2.8 % LS a
	7.7 ± 0.6 years			BA a: 2.9 % LS
Linden, *et al* (2006) (85, 86)	138 boys	Daily school physical educational classes	12 months	BMC a: +5.9 % LS
				BMD a: +2.1 % LS
	7.8 ± 0.6 years			BA a: +2.3 % LS
Specker, *et al *(2003) (87)	178 girls	High impact 30 minutes	12 months	BMC a: +9.7 % leg
	3.9 ± 0.6 years	5 times a week		
Alwis, e*t al* (2008) (19)	103 girls	Daily school physical educational classes	12 months	No effect
	7.7 ± 0.6 years			
MacKelvie, *et al*. 2004 (23)	64 boys	High impact 10-12 minutes 3 times a week	20 months	BMC a: +4.3 % FN a
	10.2 ± 0.5 years			Z a: +7.5 % FN a
Laing, *et al *(2005).(92)	143 girls	Gymnastics 1 hour once a week	24 months	BMC a: TB a, PF a
				BMD a: TB a, PF a
	6.0 ± 1.5 years			BA a: TB a PF a
Linden, *et al*. (2006) (86)	99 girls	Daily school physical educational classes	24 months	BMC a: 3.8 % LS a 3.0 % legs
				BMD a: 0.6 % TB, 1.2 % LS, 1.2 % legs
	7.6 ± 0.6 years			BA a: 1.8 % LS a, 0.3 % FN a
Alwis, *et al*. (2008) (19)	137 boys	Daily school physical educational classes	24 months	BMC a: +3.0 % LS a
	7.8 ± 0.6 years			BA a: +1.3 % LS a
Alwis, *et al*. (2008) (19)	99 girls	Daily school physical educational classes	24 months	No effect
	7.6 ± 0.6 years			
**Early pubertal (Tanner stage II-III)**				
Petit, *et al* (2002) (27)	106 girls	High impact 10-12 minutes 3 times a week	7 months	BMD a: +1.7 % Tr a, +2.6 % FN
				Z a: +4.0 % FN a
	10.5 ± 0.6 years			CT: +3.2 % FN
MacKelvi, *et al*. (2001) (93)	107 girls	High impact 10-12 minutes 3 times a week	7 months	BMC a: +1.8 % LS a
				BMD a: +1.7 % LS a, +1.6 % FN a
	10.5 ± 0.6 years			vBMD a: FN a
McKay, *et al.* (2005) (94)	124 children mean	Jumping 3 x 3 minutes 5 days a week	8 months	BMC a: +2.0 % PF, +2.7 % Tr
				BA a: +1.3 % PF a
	10.1 years			HAS variables: no effect
Iuliano-Burns, *et al*. (2003) (17)	64 girls	Moderate impact 20 minutes 3 times a week with or without calcium	8.5 months	BMC a: +2.1 % LS a, +3.0 % lower leg
	8.8 ± 0.1 years			
Heinonen, *et al* (2000) (16)	58 girls	High impact 20 minutes 2 times a week	9 months	BMC: +3.3 % LS, +4.0 % FN
	11.0 ± 0.9 years			
Morris, *et al*. (1997) (25)	71 girls	Moderate impact 30 minutes 3 times a week	10 months	BMC a: +5.5 % TB a, +5.5 % LS, +4.5 % FN a, +8.3
				% PF a
				BMD a: +2.3 % TB a, + 3.6 % LS, +10.3 % FN a,
				+3.2 % PF a
	9.5 ± 0.9 years			vBMD a: +2.9 % LS a
MacKelvie, e*t al*. (2003) (22)	75 girls	High impact 10-12 minutes 3 times a week	20 months	BMC a: +4.6 % FN a, +3.7 % LS a
	9.9 ± 0.6 years			
Lofgren, *et al* (2009) (21)	248 children	Daily school physical educational classes	48 months	BMC a: +3.0 % LS a
	7.8 ± 0.6 years			BA a: +1.3 % LS a
Detter, *et al* (2010) (14)	248 children	Daily school physical educational classes	60 months	BMC a: +3.0 % LS
	7.8 ± 0.6 years			BA a: +1.3 % LS
Macdonald, *et al *(2008) (66)	197 girls	High impact 15 minutes 5 times a week	11 months	Boys
				BMC: ± 2.7 % LS, ± 1.7 % TB
				Girls
	213 boys			BMC: ± 2.0 % FN
	10.2 ± 0.6 years			Z: ± 3.7 % FN
**Pubertal (Tanner stage IV-V)**				
Blimkie, *et al*. (1996) (34)	36 girls	Weight training 3 times 1 week	6.5 months	No effect
	16.3 ± 0.3 years			
Witzke, *et al*. (2000) (95)	53 girls	Resistance exercise 30-45 minutes 3 times a week	9 months	No effect
	14.6 ± 0.5 years			
Heinonen, *et al* (2000) (16)	68 girls	High impact 20 minutes 2 times a week	9 months	No effect
	13.3 ± 0.9 years			
Nichols, *et al.* (2001) (26)	16 girls	Resistance exercise 3 times a week	15 months	BMC a: +2.3 % FN a
	15.9 ± 0.1 years			
Stear, e*t al *(2003) (96)	144 girls	Moderate impact 45 minutes 3 times a week with or without calcium	15.5 months	BMC a: +0.8 % TB a, +1.9 % LS, +2.2 % FN a, +2.2
	17.3 ± 0.3 years			% PF a, +4.8 % Tr

^a^Abbreviations: BMC, Bone mineral content; BMD, Bone mineral density; VBMD, Volumetric bone mineral density; BA, Bone area; Z, Section modulus; Ct, Cortical thickness; TB, Total body; PF, Proximal femur; FN, Femoral neck; WT, Wards triangle; TR, Trochanter; FM, Femoral midshaft; LS, Lumbar spine

The effect of physical activity on periosteal apposition is also translated to a greater increase in bone strength than an increase in bone mass alone (43, 54, 55). Three-dimensional techniques such as the peripheral quantitative computed tomography (pQCT) and magnetic resonance imaging (MRI) have identified periosteal expansion at loaded sites in physically active individuals. For example, bone size was approximately 10 % higher in the upper limbs of young pre-pubertal gymnasts than in children on an average level of physical activity (52, 53). The arms side-to-side difference in bone size was also obvious in young pre-pubertal tennis players (43, 51). Bone may also be laid down on the endosteal surface so that the cortical thickness increases. For example, there are reports that infer that the cortical cross-sectional area can be 5 to 12 % greater in the lower limbs of young runners or gymnasts compared to controls in spite of both having the same bone size (53, 56, 57). The endosteal apposition is, however, less beneficial than the periosteal apposition if the goal is to increase bone strength, as the bone resistance to bending increases by the forth power of the radial distance (58-60).

In addition, the osteogenic response in the upper and lower limbs appears to be site-specific (53, 61). For example, endosteal apposition has also been found at the 60-70 % distal humerus, but not at the 40-50 % mid humerus in young tennis players (43, 51) and there is a different response to mechanical load in the anterior-posterior compared to the medial-lateral direction and in the proximal, mid-diaphysis and distal part of the long bones (8, 53, 54, 61, 62). If these regional discrepancies in the response to mechanical loading are the result of different types of loads in different regions, different thresholds for osteogenesis response in different regions, or different load magnitudes relative to bone size in different regions is unknown.

The strength of the bone could also be increased by the redistribution of bone mass to areas submitted to high mechanical strains. Bone strength can thus be increased by changing the shape of the bone, without an accompanying increase in bone mass or bone size, an adaptive model that has been reported in both animals (8, 44, 63, 64) and humans (45, 65, 66). Furthermore, there are also reports that infer bone mass to be transferred from unloaded to skeletal loaded parts during high intensity activity (67, 68). However, there are a range of different study designs, some of the cited studies are randomized, others non-randomized, some studies include only a few individuals. In the different studies there are different types of activities used in the intervention, different intensity levels and different frequencies advocated, different drop-out rates and different leisure time activities accepted, all facts that could influence our conclusions.

## 5. Are the Bone Mass Benefits Gained during Growth Preserved with Cessation of Exercise?

**Figure 2:**
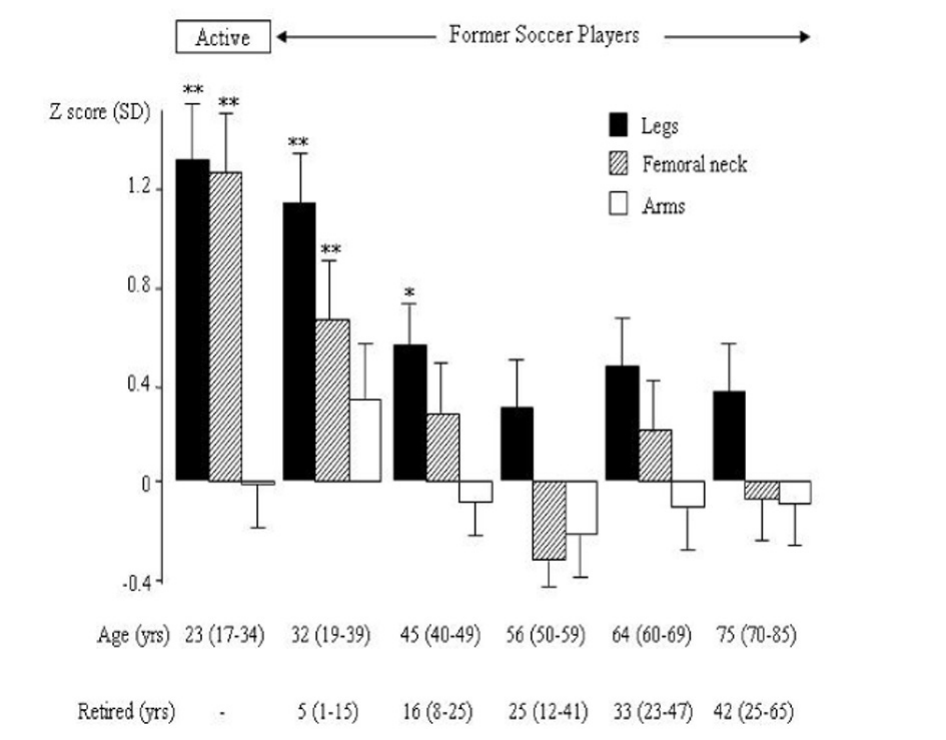
Bone Mineral Density (BMD) of the Lower Extremity, Femoral Neck and Upper Extremity in Active and Retired Male Soccer Players and Controls in Relation to Age Presented as Mean with standard deviations (SD).

**Figure 3:**
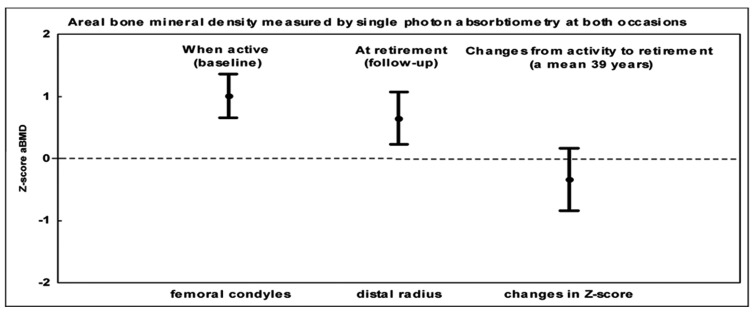
Bone Mineral Density (BMD) and Muscle Strength in 46 Male Athletes, at Baseline Median 19 (15–40) Years and 38-40 Years Later Presented as Means with 95 % Confidence Intervals.

**Figure 4:**
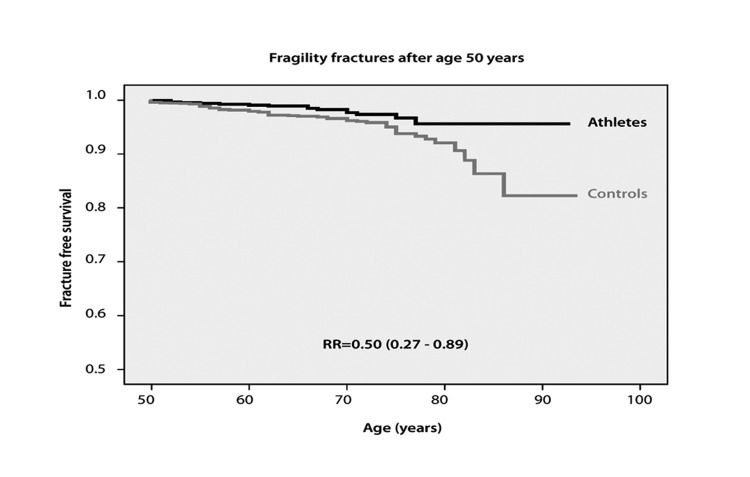
Fracture Free Survival in 709 Former Male Athletes Retired from Sports after Retirement from Sports and 1 368 Matched Controls. Rate Ratio (RR) is Presented as Mean with 95 % Confidence Interval

The exercise-induced skeletal benefits obtained during growth can, however, not be considered of clinical relevance as a preventive strategy for osteoporosis and fragility fractures unless the benefits are maintained into old age. Hypothetically, this seems less likely as the mechanostat theory indicates a decrease in bone strength as a response to reduced levels of physical activity (2, 69). Prospective studies that evaluate changes in the skeleton after a reduction in physical activity levels have been inconclusive. Seven years after an exercise intervention program in pre-pubertal children there were still significant skeletal benefits in the intervention group (70) and former elite gymnasts aged 18-35 years, who had been retired for 8 years displayed greater bone mass compared to the age-matched controls (42). This was due to a greater bone size, cortical area and trabecular volumetric density in the upper limbs and greater cortical area and trabecular volumetric density in the tibia (71). However, the residual benefits were smaller than those found during their exercise career. The same was found in prospective studies following male and female soccer players that reported that a decade after retirement from the sport, there was a greater BMD loss in the former athletes and that conferred only half of the benefits found in active athletes after 5-10 years of retirement from the sport (72, 73). Kontulainen et al. presented similar data in racket players when they reported that the dominant and non-dominant arm differences in bone mass remained after a reduction in physical activity level, but at a lower level (74). There is now also prospective, controlled study data which infers that exercise induced benefits in BMD are retained following long term retirement (Figure 2 and Figure 3). The exercise induced higher BMD in male athletes at 53-79 years of age, and after a mean of three decades of retirement from an active sports career, was still higher than expected by age, and the risk of sustaining a fragility fracture was only half when compared to the control cohort (49) (Figure 4). Unfortunately, there was no prospective structural evaluation of the skeleton performed in this report, even if cross sectional data at follow-up also inferred benefits in bone structure in favour of the former athletes. This study indicates that the faster loss in BMD found immediately after a reduction in physical activity levels could be transient.

Other reports refute the notion that exercise induced skeletal benefits are retained after an active career. This would not be unexpected, as Wolff’s law suggests that the skeleton adapts to the current level of mechanical load. Prospective data infers that there is an increased bone loss in former runners, whereas there was no loss found in those who continued with their running (75). Vuori et al. supports this view when reporting that unilateral leg presses four times a week during a 12 month period produced non-significant increases in bone mass, probably due to the relive short duration of training, but the BMD returned to pre-training levels within three months of non-training (76). Cross-sectional studies show similar results when reporting that former male soccer players have a higher residual BMD during the first two decades after retirement, but four to five decades later, only a non-significant higher leg BMD remained in the former athletes (77) (Figure 2). Virtually the same conclusions have been reported in former female soccer players (78), former male weight lifters (68, 79-81), and former male and female ballet dancers (82, 83), a common view that is now being opposed by recently published prospective controlled reports with the longest follow-up period into retirement so far (49) (Figure 3). However, most of our data in regard to the long-term effects of exercise are derived from cross-sectional studies, with the risk of being affected by secular trends in training intensity and selection bias at the baseline. Furthermore, most reported prospective studies are short-term studies that have not followed the former athletes from their active career for a maximum of three decades, that is only into ages when fragility fractures have still not become a problem of any magnitude. Also, these studies include the risk of a selection bias, as the athletes already had a higher bone mass than controls at baseline.

## 6. Are Bone Structural Benefits Gained at Growth Preserved with Cessation of Exercise?

As the mature skeleton is thought to lose bone mass essentially through remodelling on the endosteal envelope, and to a much lower extent on the periosteal envelope (84), the structural adaptations obtained by physical activity during growth periods (85-87) may be better preserved (41). This would be of clinical importance as bone structure contributes to the skeletal resistance to fractures, independently of bone mass (59). Haapasalo et al. reported an exercise-associated enlargement in bone size (humerus, radius shafts, and distal humerus) in former racket players, without a change in volumetric bone density (vBMD; g/cm^3^) which was maintained after retirement (54). Former male tennis players who had been retired for 1 to 3 years had a higher, side to side difference when comparing dominant and non dominant arms with; bone mineral content (BMC) (20 %), total cross-sectional area of bone (18 %), cortical area (22 %), bone strength index (30 %) and cortical wall thickness (15 %). The side-to-side difference in the cross-sectional area of the marrow cavity was higher at the proximal humerus (19 %) and radial shaft (29 %). These observations fit with the notion that exercise produces an enlargement of bone size that is permanent after retirement, while any endocortical thickening due to endocortical apposition may be lost or partly lost after retirement, resulting in a gradual loss in the BMD benefits. Also, in children aged 3 to 5 years, reports infer that exercise induced benefits in bone structure remained after an intervention program, at least in a short term perspective (88). There is, however, limited data in 70 to 80 year old retired athletes, the ages when fragility fractures rise exponentially. These studies suggest that the exercise-induced benefits in BMD may be eroded in those who have substantially decreased their training volumes (77), while structural benefits may persist (89). In the same cited study, the femoral neck area and lumbar spine width was reported to be larger in former male athletes, who were all above the age of 50 years and had been retired from exercise for up to 65 years, than in sedentary controls (89). Although, as the study was cross sectional, these differences could also be based on selection bias.

## 7. Are Exercise Induced Skeletal Benefits in Bone Mass Gained during Growth Preserved with Recreational Exercise?

The importance of recreational training after a high intensity training period is also supported in a variety of studies. Current training was of more importance than the previous training level in the younger years of male soccer players (77) and Huddlestone et al. reported that an arm to arm difference of 4-7 % remained in older aged former tennis players, if they continued with recreational tennis (90). These studies support the notion that recreational exercise following a period of high intensity training during younger years, may at least partly preserve exercise-induced bone mass benefits at a later stage in life.

## 8. Is Exercise during Younger Years followed by Reduced Fracture Incidence at Advanced Age?

**Figure 5:**
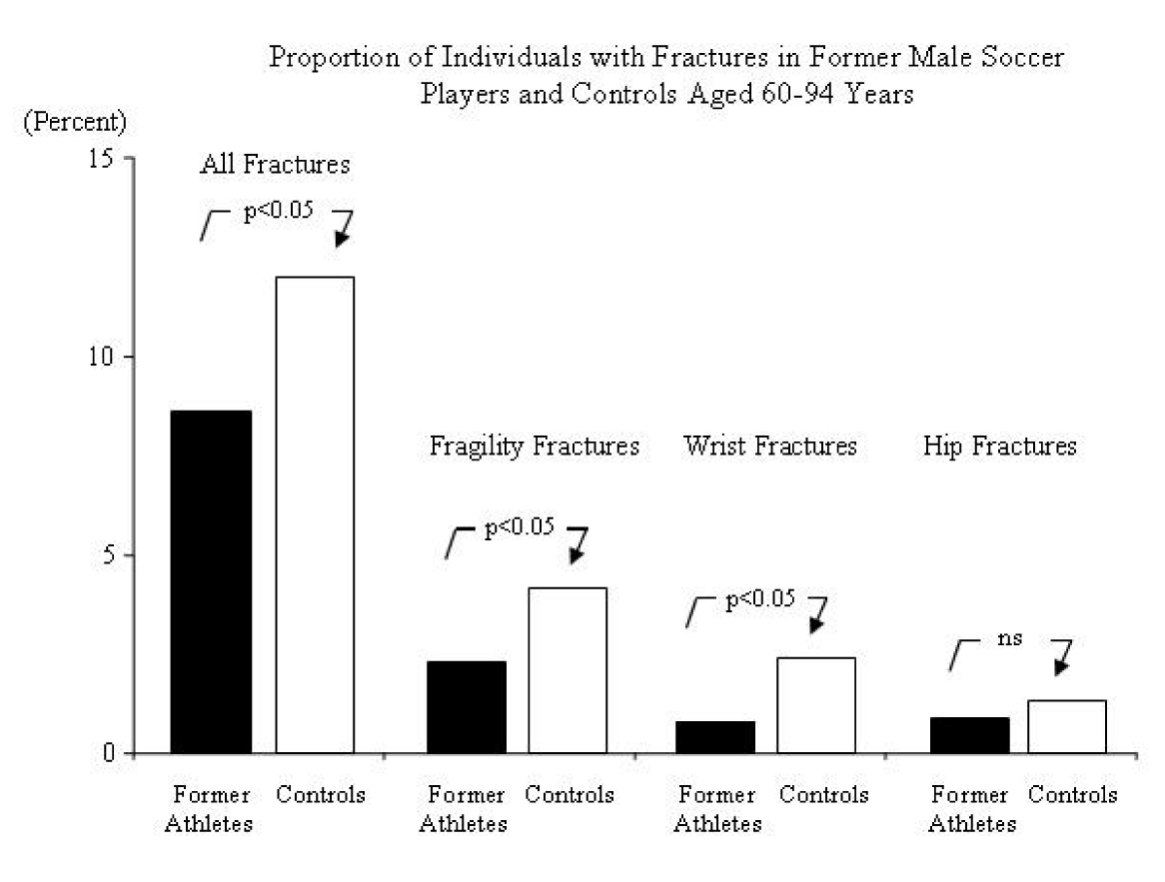
Proportion of Individuals with One or More Fractures in 663 Former Male Athletes and 943 Age and Gender Matched Controls.

If exercise induced structural skeletal benefits are retained into old age, hypothetically this ought to be associated with a lower risk of fractures than expected by age and gender. A reduced fracture risk has also been reported in retired athletes. The prevalence of fractures in 663 former athletes above the age of 50 years, and retired from sports for up to 65 years were lower than in 943 age and gender matched controls, 8.9 % in the former athletes and 12.1 % in the controls (72) (Figure 5). Additionally, the proportion of subjects with low energy fragility fractures sustained after 50 years of age was lower in the former athletes compared to the controls, 2.3 % versus 4.2 %, as well as the proportion of individuals with distal radius fractures, 0.8 % versus 2.3 %. Similar conclusions have been reported in one trial evaluating 400 former male soccer players and 800 controls (89) and now there has also been an epidemiological study published that includes 2 075 former male athletes and controls aged 50-91 years, the study reports a lower incidence of all types of fractures including fragility fractures and distal radius fractures in the former sportsmen (49) (Figure 4). But other studies refute this view. An often cited study includes 2 622 former female college athletes and 2 776 controls now aged 20-80 years, a trial that reported a similar fracture risk in both groups, 29 % versus 32 % (91). However, as this study includes individuals with as low an age as 20 years with an extremely short period of both exercise career and retirement period, and with different levels of recreational exercise after retirement, the data is difficult to interpret.

## 9. Conclusions and Perspective 

Childhood and adolescence are critical periods for the skeleton. Mechanical load has been shown during this period to be one of the best stimuli to enhance, not only bone mass, but also structural skeletal adaptations, both contributing to bone strength. Exercise prescription also includes a window of opportunity to improve bone strength in the late pre- and early peri-pubertal period. There is some evidence supporting the notion that skeletal gains obtained by mechanical load during growth are maintained at an advanced age, despite a reduction of physical activity in adulthood, and the notion that former male athletes have a lower fracture risk than expected by age, at least does not oppose the view that physical activity during growth and adolescence should be supported as one feasible strategy to reduce the future incidence of fragility fractures. However, as the conclusions discussed above are drawn predominantly from non-randomised studies and studies that include surrogate end points as bone mass, there is a need for future randomized controlled trials and studies that use the clinical relevant end-point, fractures, when evaluating if physical activity could be used as a strategy to reduce the number of fractures in older people in society.
